# The effect of laparoscopic surgery in stage II and III right-sided colon cancer: a retrospective study

**DOI:** 10.1186/1477-7819-10-89

**Published:** 2012-05-17

**Authors:** Bong-Hyeon Kye, Jun-Gi Kim, Hyeon-Min Cho, Jung Hwan Lee, Hyung-Jin Kim, Young-Jin Suh, Chung-Soo Chun

**Affiliations:** 1 St. Vincent's Hospital, The Catholic University of Korea, 3-6 Ji-dong, Paldal-gu, Suwon-Si, Gyeonggi-do, 442-723, South Korea; 2 Seoul St. Mary’s Hospital, Department of Surgery, College of Medicine, The Catholic University of Korea, 505 Banpo-dong, Seoul, Seocho-gu, 137-701, South Korea

**Keywords:** Laparoscopic surgery, Learning curve, Long-term outcome, Right sided colon cancer

## Abstract

**Background:**

This retrospective study compared the clinicopathological results among three groups divided by time sequence to evaluate the impact of introducing laparoscopic surgery on long-term oncological outcomes for right-sided colon cancer.

**Methods:**

From April 1986 to December 2006, 200 patients who underwent elective surgery with stage II and III right-sided colon cancer were analyzed. The period for group I referred back to the time when laparoscopic approach had not yet been introduced. The period for group II was designated as the time when first laparoscopic approach for right colectomy was carried out until we overcame its learning curve. The period for group III was the period after overcoming this learning curve.

**Results:**

When groups I and II, and groups II and III were compared, overall survival (OS) did not differ significantly whereas disease-free survival (DFS) in groups I and III were statistically higher than in group II (*P* = 0.042 and *P* = 0.050). In group III, laparoscopic surgery had a tendency to provide better long-term OS ( *P* = 0.2036) and DFS ( *P* = 0.2356) than open surgery. Also, the incidence of local recurrence in group III (2.6%) was significantly lower than that in groups II (7.4%) and I (12.1%) ( *P* = 0.013).

**Conclusions:**

Institutions should standardize their techniques and then provide fellowship training for newcomers of laparoscopic colon cancer surgery. This technique once mastered will become the gold standard approach to colon surgery as it is both safe and feasible considering the oncological and technical aspects.

## Background

Colorectal cancer, the second most common cancer in Korea, has been increasing exponentially probably due to Westernized dietary habits. After the first laparoscopic colon resection was reported by Jacobs *et al*. [1] in 1991 many articles were published about the feasibility of laparoscopic surgery. Then, numerous articles acclaimed laparoscopic surgery for its short-term benefits despite the long operation time. Recently, articles from multicenter trials suggested that long-term outcome of laparoscopic surgery was not inferior to that of open surgery [2-5]. Although many data are yet to be statistically validated, results tend to show that advantages of laparoscopic surgery outweigh those of open surgery [6-8]. It is imperative to overcome the learning curve (LC) of laparoscopic surgery but once conquered, laparoscopic surgery appears to be a more desirable procedure with numerous benefits. In our institution, we introduced laparoscopic surgery in 1991 and have performed laparoscopic colon surgery since 1994 to the present. This procedure has now become a standard surgical procedure for colon cancer in our institute and this has led us to observe whether long-term oncological outcome of laparoscopic colon cancer surgery was comparable to that of open surgery. Moreover, we were curious to find out the short- and long-term consequences of the LC.

At our institute we gathered 21 years of data to evaluate the long-term oncological impact of introducing the laparoscopic approach in right-sided colon cancer surgery and to propose whether laparoscopic surgery should replace open surgery in the near future and become the gold standard method in the area of colorectal surgery.

## Methods

In total, 218 patients revealing pathologic stage II or III of right-sided colon cancer who underwent curative resection at the Department of Surgery, St. Vincent’s Hospital, The Catholic University of Korea from April 1986 to December 2006 were evaluated retrospectively. Of those, 18 patients who underwent emergency operation for cancer perforation or obstruction were excluded. After obtaining the review board approval from our institute, demographics and tumor stages of 200 patients who underwent elective surgery were identified. For our study, we defined right-sided colon cancer as a confirmed adenocarcinoma arising from the cecum, ascending colon, hepatic flexure colon, or proximal transverse colon. We performed right hemicolectomy (RHC) or extended right hemicolectomy (ERHC) for right-sided colon cancer. In the case of lymph node dissection, high vessel ligation that involved ligating the vessel root of ileocolic artery and mid colic artery in ERHC or ligating the right branch of mid colic artery and the root of ileocolic artery in RHC was referred to as D3. Low vessel ligation that involved ligating the vessel around marginal artery was designated as D2.

**Figure 1:**
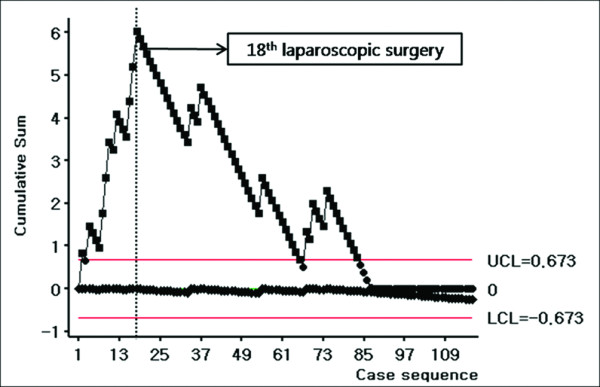
**This graph shows the learning curve (LC) for laparoscopic right-sided colon cancer surgery using CUSUM analysis (target value = 0.009).** The LCs of laparoscopic right-sided colectomy in all TNM stage had been overcome after the 18th laparoscopic right-sided colectomy.

We divided our patients into three separate groups according to time sequence. Group I referred back to the time when laparoscopic approach had not yet been introduced. Group II was designated as the time when first laparoscopic approach for right-sided colectomy was carried out until its LC was overcome. Group III was the period after overcoming this LC. For the LC of laparoscopic right-sided colectomy in our institute, we used the CUSUM model for surgical outcomes. For CUSUM charts, the observed score for successful laparoscopic surgery was defined as 0, and the score for conversion, intraoperative complications, postoperative complications, or lymph node retrieval of less than 12 from the acquired specimen from surgery were identified as 1. Also, the mean value of our series (=0.09) was used as the expected score for the CUSUM charts. With the CUSUM analysis, we found that the LC of laparoscopic right-sided colectomy in all TNM stages had been overcome after the 18th laparoscopic right-sided colectomy (Figure 1). Since the introduction of laparoscopic procedure, the same surgical methods were used even during open surgery. Thus when dividing the groups, both open and laparoscopic surgeries were included. Therefore, 58 patients enrolled in group I, 27 patients in group II, and 115 patients in group III. In the period of group II, we defined this period from the very first laparoscopic right-sided colon cancer surgery to the 18th laparoscopic operation, but this includes open surgeries as well in between. By running CUSUM analysis to obtain the LC of laparoscopic right-sided colon cancer for all TNM stages, we found that the LC was 18 cases in order to perform the surgery with a comparable level of efficiency. However, the subjects of this study were only patients with stage II and III right-sided colon cancer, so only 12 out of 18 patients were allocated to group II. Thus, in group II, 15 patients underwent open surgery and 12 patients underwent laparoscopic procedure. In group III, 34 patients underwent open surgery and 81 patients underwent laparoscopic surgery. We had kept the indication of laparoscopic colon cancer surgery for all stages except for the stage T4 cancer with severe invasion to adjacent organ. However during early period, higher costs were required for laparoscopic surgery than open surgery since laparoscopic surgery was not covered by the health insurance system in Korea at that time. For this reason, we discussed laparoscopic surgery in detail with group II patients and its high costs and whether they preferred to receive open or laparoscopic surgery. Therefore, we believe the number of patients who underwent laparoscopic surgery in group II was smaller than those who had open surgery. Since laparoscopic surgery’s benefits started to become recognized and became partially covered by the health insurance system in Korea, the number of cases increased. Still, there are patients with financial issues who cannot afford non-deductable disposable instruments such as Ligasure (Covidien, Mansfield, MA, USA) or Harmonic Scalpel (Ethicon Endo-Surgery, Cincinnati, OH, USA) opting patients to have open surgery. This describes the reason for the number of patients in group III having open surgery. We would like to clarify that with the same indication of laparoscopic or open surgery, it was the patient who decided which operative method to receive, not their disease condition. Hence, the oncological outcome was analyzed in each group. In addition, in groups II and III oncological outcome was compared between open and laparoscopic surgery.

In order to rule out unnecessary bias we needed to observe whether clinicopathological features were evenly distributed in each group. The risk factors that might have been associated with the oncological outcome were age, gender, body mass index (BMI), American Society of Anesthesiologists (ASA) physical status, tumor location, operative approach method, range of lymph node dissection, pathologic stage using UICC system, pathologic T and N status, microscopic differentiation, number of retrieved lymph nodes, whether chemotherapy was performed or not, whether reoperation was carried out or not, and preoperative or postoperative serum carcinoembryonic antigen (CEA) level.

One-way ANOVA for continuous variables and Chi-square test for categorical variables were used for comparing the clinicopathological features among three groups. Continuous variables are expressed as the mean ± standard error. The survival probability analysis was performed using the Kaplan-Meier method. The log-rank test was used to assess the difference of survival between strata. The Cox proportional-hazards regression model with forward selection was used for multivariate analysis with statistical significance accepted at P < 0.05.

The applied statistical software was SPSS® 12.0 (SPSS Inc., Chicago, IL, USA), while CUSUM model was analyzed using Minitab® 14.0 (Minitab Inc., State College, PA, USA).

## Results

The patients’ ages ranged from 25 to 87 years, with a mean age of 61.9 ± 12.6 years. A comparison of preoperative and postoperative risk factors among the three groups is shown in Table 1. As time passed, the laparoscopic approach was more frequently applied to right-sided colon cancer surgery. Becoming accustomed to the laparoscopic approach, high vessel ligation with D3 lymph node dissection was more frequently performed (*P* <0.001). Although longer operation times were needed in groups II and III than in group I, better short-term outcomes in postoperative complication and postoperative recovery were observed in group III than in group I (Table 1).

**Table 1:** Overview of clinical data in 200 patients who underwent right sided colon cancer surgery

			**Group I (*n*** **= 58)**	**Group II (*n*** **= 27)**	**Group III (*n*** **= 115)**	***P*value**
Patient characteristic	Age (years)	≤65	38 (66.7%)	16 (59.3%)	52 (45.2%)	
		>65	19 (33.3%)	11 (40.7%)	63 (54.8%)	0.007
	Gender	Male	26 (44.8%)	13 (48.1%)	56 (48.7%)	
		Female	32 (55.2%)	14 (51.9%)	59 (51.3%)	0.641
	ASA	1	41 (70.7%)	13 (48.1%)	64 (55.7%)	
		2	15 (25.9%)	13 (48.1%)	46 (40.0%)	
		3	2 (3.4%)	1 (3.7%)	5 (4.3%)	0.114
	BMI (kg/m2)	Mean ± S.D.	20.7 ± 3.9	22.4 ± 2.5	22.4 ± 3.3	0.001
Operative procedure	Operation method	RHC	45 (77.6%)	19 (70.4%)	75 (65.2%)	
		ERHC	13 (22.4%)	8 (29.6%)	40 (34.8%)	0.096
	Approach	Open	58 (100%)	15 (55.6%)	34 (29.6%)	
		Lap	-	12 (44.4%)	81 (70.4%)	<0.001
	Vessel ligation	Low	6 (26.1%)	3 (17.6%)	4 (3.7%)	
		High	17 (73.9%)	14 (82.4%)	103 (96.3%)	<0.001
Macroscopic feature	Location	Cecum	12 (20.7%)	11 (40.7%)	17 (14.8%)	
		Ascending	25 (43.1%)	9 (33.3%)	57 (49.6%)	
		H flexure	20 (34.5%)	7 (25.9%)	23 (20.0%)	
		prox T	1 (1.7%)	0	18 (15.7%)	0.097
	Tumor size	≤6.5	34 (58.6%)	17 (63.0%)	61 (53.5%)	
		>6.5	24 (41.4%)	10 (37.0%)	53 (46.5%)	0.467
		Mean ± S.D.	6.5 ± 2.6	6.6 ± 3.2	6.6 ± 2.7	0966
Microscopic feature	Stage	II	40 (69.0%)	18 (66.7%)	64 (56.1%)	
		III	18 (31.0%)	9 (33.3%)	50 (43.9%)	0.090
	Pathologic T	2	2 (3.4%)	1 (3.7%)	2 (1.8%)	
		3	47 (81.0%)	24 (88.9%)	100 (87.7%)	
		4	9 (15.5%)	2 (7.4%)	12(10.5%)	0.651
	Pathologic N	0	36 (62.1%)	18 (66.7%)	64 (56.1%)	
		1	15 (25.9%)	5 (18.5%)	31 (27.2%)	
		2	7 (12.1%)	4 (14.8%)	19 (16.7%)	0.345
	Differentiation	Well	21 (36.2%)	16 (59.3%)	17 (14.9%)	
		Moderately	19 (32.8%)	6 (22.2%)	85 (74.6%)	
		Poorly	11 (19.0%)	3 (11.1%)	9 (7.9%)	
		Mucinous	7 (12.1%)	2 (7.4%)	3 (2.6%)	0.685
	Lymphatic invasion	No	10 (35.7%)	15 (62.5%)	88 (77.2%)	
		Yes	18 (64.3%)	9 (37.5%)	26 (22.8%)	<0.001
	Venous invasion	No	25 (89.3%)	22 (91.7%)	111 (97.4%)	
		Yes	3 (10.7%)	2 (8.3%)	3 (2.6%)	0.051
	Perineural invasion	No	25 (86.2%)	22 (91.7%)	97 (85.1%)	
		Yes	4 (13.8%)	2 (8.3%)	17 (14.9%)	0.708
	Retrieved LN	<12	4 (6.9%)	10 (37.0%)	22 (19.3%)	
		≥12	54 (93.1%)	17 (63.0%)	92 (80.7%)	0.103
Additional treatment	Adjuvant CTx	No	15 (26.3%)	1 (3.8%)	17 (14.9%)	
		Yes	42 (73.7%)	25 (96.2%)	97 (85.1%)	0.105
Perioperative course	OP time(mins)	Mean ± S.D.	199.5 ± 83.4	308.5 ± 109.9	298.7 ± 86.8	<0.001
	Intraoperative Cx	No	58 (100%)	26 (96.3%)	111 (96.5%)	
		Yes	0	1 (3.7%)	4 (3.5%)	0.189
	Intraoperative TF (mL)	Mean ± S.D	361.6 ± 636.4	120.4 ± 243.9	83.3 ± 224.8	<0.001
	Postoperative Cx	No	45 (77.6%)	22 (81.5%)	102 (88.7%)	
		Yes	15 (22.4%)	5 (18.5%)	13 (11.3%)	0.052
	Postoperative TF (mL)	Mean ± S.D	156.9 ± 362.9	129.6 ± 233.8	164.5 ± 250.7	0.850
	Reoperation	No	56 (96.6%)	26 (96.3%)	114 (99.1%)	
		Yes	2 (3.4%)	1 (3.7%)	1 (0.9%)	0.223
	ICU stay (days)	mean ± S.D	4.3 ± 3.9	4.7 ± 3.0	1.9 ± 1.5	<0.001
	Hospital stay (days)	mean ± S.D	23.5 ± 11.4	17.6 ± 4.2	13.9 ± 6.9	<0.001
Serology	Preoperative CEA (ng/mL)	≤5	24 (66.7%)	18 (72.0%)	68 (65.4%)	
		>5	12 (33.3%)	7 (28.0%)	36 (34.6%)	0.790
	Postoperative CEA(ng/mL)	≤5	18 (85.7%)	13 (92.9%)	76 (85.4%)	
		>5	3 (14.3%)	1 (7.1%)	13 (14.6%)	0.818

ASA, American Society of Anesthesiologists; BMI, Body mass index; CEA, Carcinoembryonic antigen; CTx, Chemotherapy; Cx, Complication; ERHC, Extended right hemicolectomy; H flexure, Hepatic flexure; ICU, Intensive care unit; Lap, Laparoscopic; LN, Lymph node; prox T, Proximal transverse; RH, Right hemicolectomy; TF, Transfusion.

**Figure 2:**
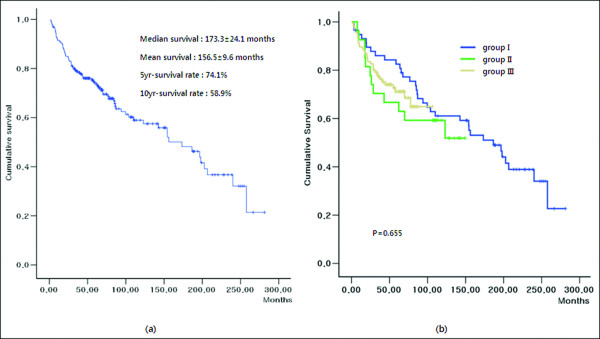
**(a) The overall survival rate of stage II and III right-sided colon cancer patients in this study.** ( **b**) Differences of overall survival rate among the three groups.

**Figure 3:**
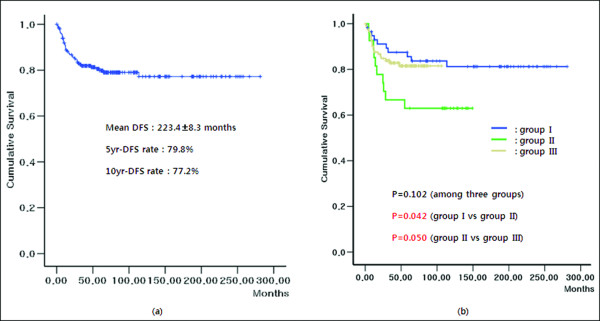
**(a) The disease-free survival rate of stage II and III right-sided colon cancer patients in the present study.** ( **b**) Differences of disease-free survival rate among the three groups. The disease-free survival rates of groups I and III were statistically higher than that of group II ( *P* = 0.042 and *P* = 0.050).

Figure 2a shows the overall survival (OS) rate of all patients in the present study. The mean OS was 156.5 ± 9.6 months. The 5- and 10-year OS rates were 74.1% and 58.9%, respectively. There was no significant difference in OS among the three groups (*P* = 0.655, Figure 2b). Unfavorable risk factors that influenced OS were advanced stage, deep invasion and lymph node involvement, lymphovascular and perineural invasion, less than 12 lymph nodes retrieval, no adjuvant chemotherapy, reoperation, and higher level of pre- and postoperative CEA. Figure 3a shows the disease-free survival (DFS) rate of all patients in the present study. The mean DFS was 223.4 ± 8.3 months. The 5- and 10-year DFS rates were 79.8% and 77.2%, respectively. There was no significant difference in DFS among the three groups (*P* = 0.102). However, when DFS between two groups were compared, the DFS rate in group II was lower than that in group I ( *P* = 0.042) and group III ( *P* = 0.050) (Figure 3b). Unfavorable factors influencing DFS were advanced stage, deep invasion and lymph node involvement, lymphovascular and perineural invasion, less than 12 lymph nodes retrieval, and higher level of preoperative CEA. In multivariate analysis, lymphatic invasion (*P* = 0.005 and *P* < 0.001), venous invasion ( *P* = 0.001 and *P* < 0.001), preoperative serum CEA level ( *P* = 0.031 and *P* < 0.001), and the retrieval of <12 lymph nodes ( *P* = 0.031 and *P* < 0.001) were independent risk factors associated with OS and DFS in stage II and III right-sided colon cancer. Of these, venous invasion was the most potent independent risk factor associated with both OS and DFS (Table 2).

**Table 2:** Univariate and multivariate analysis for overall survival and disease-free survival in this study

		**Overall survival**					**Disease-free survival**				
		**3-yr- (%)**	**5-yr (%)**	**10-yr (%)**	***P*value**		**3-yr (%)**	**5-yr (%)**	**10-yr (%)**	***P*value**	
					**Univariate**	**Multivariate**				**Univariate**	**Multivariate**
Group	I	86.1	82.5	61.1			87.5	85.6	81.2		
	II	70.3	66.7	59.3			66.7	62.9	62.9		
	III	77.2	71.2	64.9	0.655	NS	82.8	81.5	81.5	0.102	NS
Location	Cecum	82.1	79.5	63.4			84.6	78.6	78.6		
	Ascending	79.1	71.9	57.8			81.8	80.3	73.9		
	Hepatic flexure	77.9	75.6	59.4			81.3	81.3	81.3		
	Proximal transverse	73.7	66.9	58.6	0.736	NS	78.6	78.6	78.6	0.968	NS
Approach	Open	78.3	73.4	57.5			81.6	78.4	75.3		
	Lap	79.4	74.8	66.3	0.884		82.2	82.2	82.2	0.518	NS^§^
Vessel ligation	Low ligation	76.9	69.2	55.4			69.2	69.2	69.2		
	High ligation	78.9	74.1	63.8	0.834	NS	83.1	83.1	83.1	0.185	NS
Stage	II	87.6	81.8	66.6			89.1	87.9	83.9		
	III	64.8	61.6	45.5	0.004	NS	69.9	66.2	66.2	<0.001	NS
Pathologic T	2	60.0	60.0	60.0			60.0	60.0	60.0		
	3	82.4	78.2	62.3			84.3	82.6	80.5		
	4	56.2	45.4	31.1	0.003	NS	67.3	60.6	52.9	0.015	NS
Pathologic N	0	87.3	81.3	66.9			89.6	88.3	84.1		
	1	72.4	70.1	48.2			80.9	80.9	80.9		
	2	55.2	50.6	43.4	0.008	NS	53.1	44.6	44.6	<0.001	NS
Differentiation	Well	85.2	79.5	68.5			88.4	84.4	80.7		
	Moderately	79.9	75.1	50.8			83.3	83.3	83.3		
	Poorly	60.9	56.2	45.4			65.9	59.9	53.9		
	Mucinous	72.7	72.7	60.6	0.091	NS	66.7	66.7	66.7	0.025	NS
Lymphatic invasion	No	83.9	79.6	63.6			87.4	87.4	87.4		
	Yes	64.2	59.7	45.3	0.004	0.005	65.4	63.2	63.2	<0.001	<0.001
Venous invasion	No	79.7	75.1	58.4			83.6	82.8	82.8		
	Yes	25.0	25.0	25.0	<0.001	0.001	12.5	12.5	12.5	<0.001	<0.001
Perineural invasion	No	80.5	77.1	59.1			82.9	82.9	82.9		
	Yes	56.5	46.3	46.3	0.029	NS	62.2	55.9	55.9	0.006	NS
Lymph node retrieval	<12	58.3	52.5	45.9			62.8	62.8	62.8		
	≥12	83.3	78.8	62.3	0.012	0.031	85.9	83.4	80.5	0.002	<0.001
Adjuvant CTx	No	69.7	62.1	36.5			82.6	82.6	82.6		
	Yes	80.3	76.1	65.1	0.008	NS	81.4	79.0	75.9	0.621	NS
Reoperation	No	79.4	74.5	60.1			82.1	79.9	77.4		
	Yes	50.0	50.0	25.0	0.041	NS	75.0	75.0	75.0	0.463	NS
Preoperative CEA	≤5	89.1	83.5	68.1			89.8	87.5	82.3		
	>5	64.7	62.5	49.1	0.003	0.031	68.2	65.2	65.2	<0.001	<0.001
Postoperative CEA	≤5	83.1	77.8	65.1			82.8	80.0	75.8		
	>5	52.9	52.9	52.9	0.018	NS	64.2	64.2	64.2	0.051	NS

CEA, Carcinoembryonic antigen; CTx, Chemotherapy; NS, Not significant.

**Figure 4:**
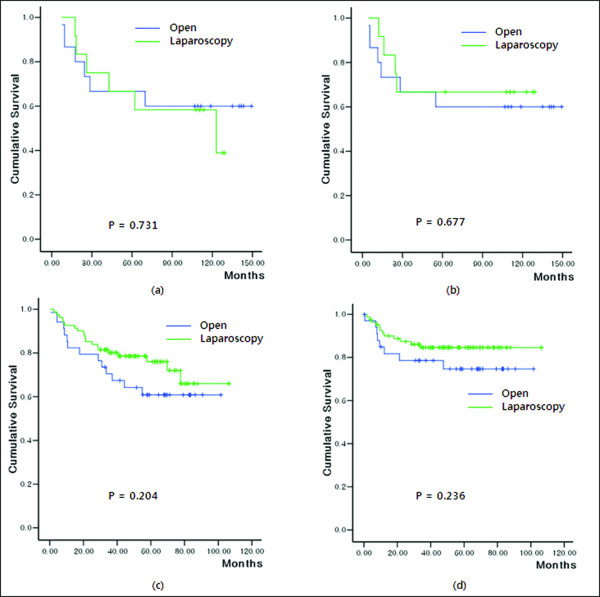
**These graphs show the difference of overall survival (OS) and disease-free survival (DFS) between the open surgery group and the laparoscopic surgery group according to the time sequence.** ( **a**) and ( **b**) are graphs for OS and DFS, respectively, in group II; ( **c**) and ( **d**) in group III.

For identifying the safety of laparoscopic surgery, in groups II and III oncological outcome was compared between the open and laparoscopic surgery groups. In group II, comparison between the laparoscopic surgery group and the open surgery group showed no difference in OS (*P* = 0.731) and DFS ( *P* = 0.677) was observed (Figure 4a and Figure 4b). However, in group III, although not significant statistically, the laparoscopic surgery group had a tendency to provide better long-term OS (*P* = 0.204) and DFS ( *P* = 0.236) than the open surgery group (Figure 4c d).

Table 3 shows the univariate and multivariate analysis for risk factors of local recurrence in our series. All local recurrences in our series occurred within 60 months after surgery for primary lesion. Since the application of the laparoscopic approach for right-sided colon cancer surgery, the incidence of local recurrence decreased (*P* = 0.013). The incidence of local recurrence was significantly lower in patients who underwent high vessel ligation ( *P* = 0.032) and the retrieval of more than 12 lymph nodes ( *P* = 0.044). Also, poorly differentiated tumor ( *P* = 0.042) and venous invasion ( *P* = 0.049) were worse prognostic factors for local recurrence. In multivariate analysis, the level of vessel ligation (the range of lymph node dissection) was the only independent risk factor for local recurrence.

**Table 3:** Analysis for risk factor of local recurrence in this study

		**No (*n*** **= 188)**	**Yes (*n*** **= 12)**	**Univariate**	**Multivariate**
				***P*value**	***P*value**
Group	I	51 (87.9%)	7 (12.1%)		
	II	25 (92.6%)	2 (7.4%)		
	III	112 (97.4%)	3 (2.6%)	0.013	0.304
Location	Cecum	36 (90.0%)	4 (10.0%)		
	Ascending	87 (95.6%)	4 (4.4%)		
	Hepatic flexure	47 (94.0%)	3 (6.0%)		
	Proximal transverse	18 (94.7%)	1 (5.3%)	0.525	
Approach	Open	98 (91.6%)	9 (8.4%)		
	Lap	90 (96.8%	3 (3.2%)	0.146	
Vessel ligation	Low ligation	11 (84.6%)	2 (15.4%)		
	High ligation	130 (97%)	4 (3.0%)	0.032	0.019
Stage	II	117 (95.9%)	5 (4.1%)		
	III	70 (90.9%)	7 (9.1%)	0.220	
Pathologic T	2	5 (100%)	0		
	3	162 (94.7%)	9 (5.3%)		
	4	20 (87.0%)	3 (13.0%)	0.118	
Pathologic N	0	113 (95.8%)	5 (4.2%)		
	1	46 (90.2%)	5 (9.8%)		
	2	28 (93.3%)	2 (6.7%)	0.355	
Differentiation	Well	53 (98.1%)	1 (1.9%)		
	Moderately	104 (94.5%)	6 (5.5%)		
	Poorly	19 (82.6%)	4 (17.4%)		
	Mucinous	11 (91.7%)	1 (8.3%)	0.042	0.571
Lymphatic invasion	No	108 (95.6%)	5 (4.4%)		
	Yes	50 (94.3%)	3 (5.7%)	0.711	
Venous invasion	No	152 (96.2%)	6 (3.8%)		
	Yes	6 (75.0%)	2 (25.0%)	0.049	0.643
Perineural invasion	No	137 (95.1%)	7 (4.9%)		
	Yes	22 (95.7%)	1 (4.3%)	0.915	
Lymph node retrieval	<12	31 (86.1%)	5 (13.9%)		
	≥12	156 (95.7%)	7 (4.3%)	0.044	0.995
Adjuvant CTx	No	31 (93.9%)	2 (6.1%)		
	Yes	154 (93.9%)	10 (6.1%)	0.994	
Reoperation	No	184 (93.9%)	12 (6.1%)		
	Yes	4 (100%)	0	0.611	
Preoperative CEA	≤5	105 (95.5%)	5 (2.5%)		
	>5	51 (92.7%)	4 (7.3%)	0.483	
Postoperative CEA	≤5	102 (95.3%)	5 (4.7%)		
	>5	16 (94.1%)	1 (5.9%)	0.595	

CEA, Carcinoembryonic antigen; CTx, Chemotherapy.

## Discussion

Laparoscopic colon resection has advantages over open resection in terms of improved early surgical outcomes, including less need for analgesia, earlier resumption of eating, shorter length of hospital stay, and lower rate of postoperative morbidity [6-8]. However, in cancer surgery, the long-term oncological outcomes appear to be more important than perioperative outcomes. It has been shown in several prospective randomized controlled trials that the long term oncological outcome of laparoscopic surgery in colon cancer is not inferior to that of open surgery [4,5,8]. Yet, many institutions still contemplate whether or not to standardize laparoscopic surgery for colon cancer. This may be due to the steep LC posing as a major challenge for surgeons with also the fear that the LC will jeopardize the oncological outcome during the time needed to polish their craft [9,10].

In respect of long-term oncological outcome, prognostic factors are divided into two categories: factors related to the tumor itself; and factors relating to the surgical procedure. Factors related to the tumor itself are depth of invasion, lymph node metastasis, tumor differentiation, lymphovascular invasion, and preoperative serum CEA level. Factors related to the surgical procedure are adequate resection margin, radical lymphadenectomy, and adequate lymph node harvest. Perioperative complications and failure of successful laparoscopic procedure such as conversion are also factors related to the surgical procedure. In our study, within the factors related to the tumor, we observed that lymphatic invasion (*P* = 0.005 and *P* < 0.001), venous invasion ( *P* = 0.001 and *P* < 0.001), and preoperative serum CEA level ( *P* = 0.031 and *P* < 0.001) were independent risk factors associated with OS and DFS in stage II and III right-sided colon cancer. For patients with above poor prognostic factors, adjuvant therapy such as chemotherapy may be helpful to improve long-term oncological outcomes. Within the factors related to the surgical procedure, adequate lymph node harvest was the only independent prognostic factor related to the surgical procedure ( *P* = 0.031 and *P* < 0.001) in both OS and DFS.

The number of harvested lymph nodes may be related to not only the knowledge of anatomy and the experience of surgeon but also the accurate pathologic reporting system. The number of examined lymph nodes itself has prognostic value in predicting outcome. The survival of patients with stage II and III colon cancer has increased with the increased number of lymph nodes examined [11]. In the present study, adequate lymph node harvest was the factor in measuring the LC therefore inadequate lymph node harvest was more frequently found in group II (37.0%) than in group I (6.9%) or group III (19.3%) (Table 1). These differences in nodal counts may have risen from inadequate lymphadenectomy by a surgeon during the LC period. Also, the DFS rate in group II was worse than in group I and III (*P* = 0.042 and *P* = 0.050, respectively) (Figure 3b). It remains to be seen whether differences in nodal counts among the three groups are clinically meaningful but from the data gathered the LC period with fewer lymph nodes retrieved seems to correlate with low DFS rate.

Ptock *et al*. [12] demonstrated that conversion of laparoscopic colon cancer resection worsens DFS in locally advanced stage II carcinoma. They especially emphasized the effect of experience of a surgeon on a successful laparoscopic colon cancer resection. A few recent studies showed that postoperative complication such as anastomotic leakage was associated with poor survival [13-15]. Tsuchiya *et al*. [16] demonstrated that factors such as excessive bleeding, lengthening of operation time, and an increase of surgical manipulation would clearly induce surgical stress. They suggested that several responses induced by surgical stress, such as neuroendocrine responses, cytokine responses, metabolic responses, and other yet unknown biologic responses, may result in a marked enhancement of tumor metastasis by affecting residual or circulating cancer cells or normal host cells in the target organ or tissue of metastasis. In the period of the LC, the condition induced to increase the surgical stress such as conversion or perioperative complication may contribute to DFS negatively. Also, considering the immunological effect, longer operation time in group II may affect DFS negatively [17,18]. Kang *et al*. [19] demonstrated that technical difficulty during laparoscopy-assisted surgery jeopardizes oncological safety. They insisted that for oncological safety following laparoscopic assisted surgery, technical difficulty and the number of procedures performed should be considered to evaluate the learning process for a laparoscopic surgeon. In the early period of our laparoscopic series, we experienced many technical difficulties. These technical difficulties resulting from inexperience also may have affected DFS negatively. Lessening of these risk factors related to surgical procedure may be conceivably possible by the endeavor of the surgeons.In the LC period, although surgeons tried to maintain the oncological principle, the no-touch isolation technique fell short in some surgeries, and factors such as conversion rate, operation time, and perioperative complications increased in others [9,10,20]. These factors may all have contributed to the increase in surgical stress but despite these setbacks, through perseverance and exploration of numerous techniques, we managed to standardize right-sided colon cancer surgery using the laparoscopic approach. Firstly, right colic mesentery was lifted from the retro-peritoneum without touching the colon itself, focusing on dissecting the mesentery with the diseased colon. For right colectomy, the ileocolic artery and vein, the right-sided branch of the midcolic artery and vein, and the right colic artery and vein if present were skeletonized from their original sites. They were clipped three times at their original sites and cut between the distal second and third clip. This allowed the diseased colon and mesentery to be successfully isolated from the superior mesenteric artery and vein without injuring or touching other parts of the colon. Hence, high vessel ligation (D3 lymph node dissection) was more frequently performed after the introduction of laparoscopic surgery (Table 1). This division prior to tumor-containing bowel mobilization could minimize cancer cell spread though the draining vessels. The medial to lateral approach may be more aligned with the no-touch principle than the lateral to medial approach. Recently, some reports demonstrated that progress in laparoscopic techniques for colorectal cancer surgery has resulted in the development of a medial to lateral approach for D3 lymphadenectomy [21,22]. Compared with the classical lateral to medial approach, D3 lymphadenectomy using the medial to lateral has the advantage of abiding by the no-touch isolation technique for oncologic surgery [21]. Since Turnbull *et al.*[23] described the advantage of the no-touch isolation technique for cancer surgery, many reports demonstrated better oncological outcome with this technique [24-26]. Hayashi *et al*. [26] demonstrated that the no-touch isolation technique could prevent cancer cells from being shed into the portal circulation during surgical manipulation. Although exact statistical value was not measured, approach for lymphadenectomy had been mixed to ‘lateral to medial approach’ and ‘medial to lateral approach’ in group I due to different policies amongst several surgeons. Since laparoscopic surgery was introduced, medial to lateral approach became the standard procedure for D3 lymphadenectomy in open as well as laparoscopic colorectal cancer surgery in our institution. In the present study, with gradually increased high vessel ligation, the local recurrence rate decreased gradually in open as well as in laparoscopic surgery after the introduction of laparoscopic surgery (*P* = 0.013). Also, in multivariate analysis, we found that high vessel ligation was the only independent prognostic factor for local recurrence ( *P* = 0.019) (Table 3). This finding appears to result from the endeavor of standardizing the surgical procedure, including the accomplishment of D3 lymphadenectomy through high vessel ligation and the medial to lateral approach whilst experiencing the LC in laparoscopic surgery. With the tendency of better long-term oncological outcome of laparoscopic surgery shown in Figure 4, this finding may be considered as the advantage of laparoscopic surgery through the standardization of the technique.

In our study, two surgeons performed laparoscopic surgery. The main surgeon, a pioneer in laparoscopic abdominal surgery in Korea, began performing laparoscopic right sided colon cancer surgery in April 1995. Another surgeon, a junior surgeon, who had experienced laparoscopic surgery as a scope operator for main surgeon’s laparoscopic procedure beginning in March 2001, began laparoscopic right sided colon cancer surgery in April 2004. The LC was based on the results of the main surgeon alone as the corresponding author, (J-G Kim) specializing in colorectal surgery, performed most of the right sided colon cancer surgeries. Although it is unpublished data, we could observe that the LC of the junior surgeon did not have an influence on the oncological outcome. This was because the junior surgeon began his laparoscopic procedure under supervision of a pioneer surgeon after the standardization of the procedure was complete. As shown in Figure 4 and Table 3, long-term oncological outcomes of laparoscopic surgery group in group II are not inferior to the open surgery group. In group III, long-term oncological outcomes of laparoscopic surgery group showed better outcome than the open surgery group. Some reports suggested that the standardization of the colorectal cancer surgery would result in not only the reduction of the LC period but also in lowering recurrence rate and better OS [10,27]. In our study, while the procedure during the LC resulted in the negative effect on DFS, we observed that once the standardization of the procedure was complete laparoscopic surgery had a tendency to provide reduction in recurrence rate and improved long term oncological outcome compared to open surgery.

Our study has limitations in that it was a retrospective study using the medical records of patients, and the follow-up observation could not be performed in all patients. However, while most reports for the LC of laparoscopic surgery suggested perioperative short-term outcomes as factors for measuring the LC of laparoscopic surgery, our study found that surgical and pathological factors must be considered to measure the LC for cancer surgery.

## Conclusions

The laparoscopic approach for stage II and III right-sided colon cancer is safe and feasible in both oncological and technical aspects. However, because of the probability of poor oncological outcomes during the LC period, surgeons must make great efforts to shorten this time frame. Institutions should standardize their techniques and then provide fellowship training for newcomers of laparoscopic colon cancer surgery as this technique once mastered will become the gold standard approach to colon surgery.

## Competing interests

The manuscript is an original work and has not been submitted or is under consideration for publication in another journal. The study complies with current ethical consideration. We also confirm that all the listed authors have participated actively in the study, and have seen and approved the submitted manuscript. The authors do not have any possible conflicts of interest. No competing financial interests exist.

## Authors’ contributions

K and JGK conceived and designed the study; K, C, HJK, L, S, JGK, and C acquired the data; K, C, HJK, L, S, JGK, and C analyzed and interpreted the data; K, HJK, JGK, and C drafted the manuscript; K, C, HJK, JGK, S, L, and C critically revised the manuscript; K, C, HJK, L, S, JGK, and C approved the final manuscript. All authors read and approved the final manuscript.
